# Apixaban Dose in Patients With Atrial Fibrillation and Acute Coronary Syndrome and/or Undergoing Percutaneous Coronary Intervention: Insights From AUGUSTUS

**DOI:** 10.1016/j.jacadv.2025.101665

**Published:** 2025-03-20

**Authors:** Marat Fudim, Renato D. Lopes, Daniel M. Wojdyla, Roxana Mehran, Muhammad Shahzeb Khan, Christopher B. Granger, Shaun G. Goodman, Ronald Aronson, Stephan Windecker, John H. Alexander

**Affiliations:** a Duke Clinical Research Institute, Duke University School of Medicine, Durham, North Carolina, USA; b Zena and Michael A. Wiener Cardiovascular Institute, Icahn School of Medicine at Mount Sinai, and Cardiovascular Research Foundation, New York, New York, USA; c Canadian VIGOUR Centre, University of Alberta, Edmonton, Canada; d Terrence Donnelly Heart Centre, St. Michael's Hospital, University of Toronto, Toronto, Canada; e Bristol Myers Squibb, Princeton, New Jersey, USA

**Keywords:** acute coronary syndrome(s), apixaban, atrial fibrillation, dose reduction, percutaneous coronary intervention

## Abstract

**Background:**

Studies have demonstrated the safety and efficacy of reducing the dose of apixaban from 5.0 mg to 2.5 mg twice daily in patients with atrial fibrillation (AF) and ≥2 dose-reduction criteria (age ≥80 years, body weight ≤60 kg, serum creatinine ≥1.5 mg/dL). However, data on reduced dose apixaban in patients with AF and acute coronary syndrome (ACS) and/or percutaneous coronary intervention (PCI) are limited.

**Objectives:**

The authors aimed to assess clinical outcomes, including bleeding and death/ischemic events, according to apixaban dose in AUGUSTUS.

**Methods:**

In AUGUSTUS, 4,614 patients with AF and/or recent ACS or PCI on a P2Y12 inhibitor were randomized to open-label apixaban or vitamin K antagonist (VKA) and blinded aspirin or placebo for 6 months. Apixaban dose was determined by investigators following the apixaban label. We assessed outcomes, including major/clinically relevant nonmajor bleeding and death/ischemic events, among patients who appropriately received reduced dose apixaban, inappropriately received reduced dose apixaban, and appropriately received standard dose apixaban compared with VKA.

**Results:**

Of 2,290 patients assigned apixaban, 229 (10%) received reduced dose apixaban and 98 (43%) of those met dose-reduction criteria. Among patients receiving appropriately reduced, inappropriately reduced, and standard dose apixaban, rates of major/clinically relevant nonmajor bleeding were 13.7%, 10.5%, and 11.0%; rates of death or ischemic events were 12.2%, 12.3%, and 5.7%. When comparing the risk of clinical outcomes in the 3 groups (appropriately reduced, inappropriately reduced, and standard dose apixaban) vs matched patients receiving VKA, we found that patients receiving apixaban had more favorable outcomes than those receiving VKA, without significant interaction (*P* > 0.2 across all 3 groups and all outcomes).

**Conclusions:**

Of the ∼10% of patients in AUGUSTUS who received reduced dose apixaban, less than half met the dose-reduction criteria. In patients with AF and recent ACS or PCI, appropriately reduced dose apixaban was associated with a lower risk of bleeding and similar rates of ischemic outcomes compared with VKA, similar results were found with standard dose apixaban. (A Study of Apixaban in Patients With Atrial Fibrillation, Not Caused by a Heart Valve Problem, Who Are at Risk for Thrombosis [Blood Clots] Due to Having Had a Recent Coronary Event, Such as a Heart Attack or a Procedure to Open the Vessels of the Heart; NCT02415400)

Approximately half of those with atrial fibrillation (AF) have accompanying coronary artery disease, and roughly 10% will require percutaneous coronary intervention (PCI).^1^, ^2^, ^3^ Patients with AF who have had an acute coronary syndrome (ACS) are at a higher risk of adverse outcomes, including mortality, than patients without AF.^4^^,^^5^ It can be challenging to identify an antithrombotic regimen for patients with AF and ACS that takes into consideration both bleeding and ischemic events.^6^^,^^7^ Antithrombotic therapy in these patients usually includes an oral anticoagulant, which can be either a vitamin K antagonist (VKA) (ie, warfarin) or a direct oral anticoagulant, and dual antiplatelet therapy consisting of aspirin and a P2Y12 inhibitor.^7^^,^^8^

Often, a combination of a direct oral anticoagulant and dual antiplatelet therapy (ie, triple therapy) is used in patients with AF and ACS. Triple therapy is associated with a high rate of significant bleeding compared with dual or single antithrombotic therapy.^8^, ^9^, ^10^ Antiplatelet and anticoagulation management in patients with AF and a recent ACS and/or undergoing PCI was assessed in AUGUSTUS (An Open-Label, Prospective, Multicenter, 2×2 Factorial, Randomized Controlled, Clinical Trial to Evaluate the Safety of Apixaban vs VKA and Aspirin vs Aspirin Placebo in Patients with Atrial Fibrillation and Acute Coronary Syndrome or Percutaneous Coronary Intervention). Results from AUGUSTUS demonstrated that bleeding and death or hospitalization were significantly lower, without substantial increases in ischemic outcomes, with a regimen including apixaban without aspirin than with regimens including VKA, aspirin, or both.^11^

Previous studies have demonstrated that a reduced dose of apixaban of 2.5 mg twice daily is safe and efficacious in patients with AF and ≥2 of the following criteria: age ≥80 years, body weight ≤60 kg, and serum creatinine ≥1.5 mg/dL (133 μmol/L).^12^^,^^13^ However, there are no data regarding the use of reduced vs standard dose apixaban in patients with AF and a recent ACS and/or PCI or in other clinical settings in which triple therapy is indicated. In this post hoc analysis of the AUGUSTUS trial, we determined the number of patients who received reduced dose apixaban and the proportion who met the specified dose-reduction criteria. We then assessed the outcomes of patients who appropriately or inappropriately received reduced dose apixaban, or appropriately received standard dose apixaban. We then compared the outcomes of patients appropriately receiving reduced or standard dose apixaban with similar patients receiving VKA.

## Methods

### Study design and patient sample

The design, rationale, and results of AUGUSTUS have been published. AUGUSTUS was an open-label, prospective, multicenter, 2×2 factorial design clinical trial that compared apixaban with a VKA and aspirin with placebo in patients with AF who had a recent ACS and/or underwent PCI.^11^ The trial enrolled 4,614 patients who were ≥18 years of age with a history of persistent, permanent, or paroxysmal AF with planned long-term use of an oral anticoagulant; recent ACS and/or PCI; and planned use of a P2Y12 inhibitor for at least 6 months. Exclusion criteria included use of oral anticoagulation for any other condition (eg, prosthetic valves, venous thromboembolism, and mitral stenosis); severe renal insufficiency (estimated creatinine clearance <30 mL/min); history of intracranial hemorrhage; recent or planned coronary artery bypass graft surgery; coagulopathy or current bleeding; and contraindication to a VKA, apixaban, P2Y12 inhibitors, or aspirin. Patients were followed for 6 months. The study was approved by the individual site's Institutional Review Board or a central Institutional Review Board. Patients signed an informed consent upon enrollment into the original clinical trial.

### Randomization and interventions

Patients were randomized to receive open-label apixaban or VKA and blinded aspirin or matching placebo within 14 days after having an ACS and/or undergoing PCI. Randomization was stratified according to ACS or elective PCI at enrollment.

Apixaban dose was determined by investigators according to the apixaban label. The suggested apixaban dosing was 5.0 mg twice daily or 2.5 mg twice daily if patients had ≥2 of the following criteria: age ≥80 years, body weight ≤60 kg, or serum creatinine ≥1.5 mg/dL (133 μmol/L). Patients randomized to VKA had the dose adjusted to reach a target international normalized ratio of 2.0 to 3.0. For the comparison of aspirin with placebo, patients received aspirin 81 mg or matching placebo once daily. The treatment duration for all regimens was 6 months, during which the use of other anticoagulants (eg, dabigatran, rivaroxaban, edoxaban, unfractionated heparin, or low-molecular-weight heparins) was prohibited.

### Clinical outcomes

The primary outcome was the incidence of major or clinically relevant nonmajor (CRNM) bleeding as defined by the International Society on Thrombosis and Haemostasis.^14^ Major bleeding was defined as bleeding resulting in death; occurring in a critical organ (intracranial, intraspinal, intraocular, retroperitoneal, intraarticular, intramuscular with compartment syndrome, or pericardial); or associated with a decrease in hemoglobin level of ≥2 g/dL; or requiring transfusion of ≥2 U of packed red blood cells. CRNM bleeding was defined as bleeding resulting in hospitalization, medical or surgical intervention, an unscheduled clinic visit, or a change in physician-directed antithrombotic therapy.^15^

Secondary outcomes included the composite of death or hospitalization and the composite of death or an ischemic event including stroke, myocardial infarction, probable or definitive stent thrombosis, or urgent revascularization. Other outcomes included individual components of the secondary outcomes. All bleeding and ischemic events (except for urgent revascularization) were independently adjudicated by a clinical events classification committee whose members were blinded to study drug assignment.^12^^,^^13^

### Statistical analysis

Cross-tabulations were used to demonstrate both agreement between meeting the dose-reduction criteria and receiving a reduced dose of apixaban, and the number of dose-reduction criteria met and randomized treatment received. To describe the baseline characteristics according to apixaban dose, we classified patients assigned to apixaban into 4 groups: 1) met criteria for dose reduction and received a reduced dose (appropriately received reduced dose); 2) met criteria for dose reduction and received a standard dose (inappropriately received standard dose); 3) did not meet criteria for dose reduction and received a reduced dose (inappropriately received reduced dose); and 4) did not meet criteria for reduced dose and received a standard dose (appropriately received standard dose). To create a matched comparator, patients receiving VKA were classified into 2 groups based on the applicable apixaban dose reduction criteria: 1) met criteria for dose reduction; and 2) did not meet criteria for dose reduction. Due to the small number of patients (n = 8), those who met criteria for dose reduction and received standard dose apixaban (inappropriately receiving standard dose) were not included in the current analysis. Continuous characteristics were summarized as median and quartiles or mean and SD and categorical characteristics were presented as frequencies and percentages. Patients in the apixaban group who met the criteria for dose reduction and received a reduced dose were compared with patients in the VKA group who met the criteria for dose reduction. Additionally, we compared patients in the apixaban group who appropriately received the reduced dose vs patients who inappropriately received the standard dose. For continuous variables, Wilcoxon and *t*-test were used while for categorical variables chi-square tests were applied. For comparison with small sample size, exact Pearson chi-square tests were applied.

We then assessed rates of major or CRNM bleeding, death or hospitalization, and death or ischemic events among patients appropriately receiving reduced dose apixaban, inappropriately receiving reduced dose apixaban, and appropriately receiving standard dose apixaban using Kaplan-Meier estimates at 6 months. Bleeding events were included from the first dose of study drug to 6 months, permanent study drug discontinuation, or death; hospitalization and ischemic events were included from randomization to 6 months. We compared the event rates across the 3 groups using the log-rank test.

Finally, we compared rates of the same outcomes among patients who appropriately received reduced dose apixaban with similar patients receiving VKA and among patients who received standard dose apixaban with similar patients receiving VKA. Among patients receiving apixaban who did not meet the dose-reduction criteria, a propensity score model was developed to estimate the probability of receiving reduced dose vs standard dose. The estimated model was then applied to the patients receiving VKA who did not meet dose-reduction criteria and patients were matched (apixaban/VKA) based on the predicted probability of receiving a reduced dose. Exact matching was required for sex and the 3 dose-reduction criteria (age, weight, and creatinine). Treatment effects were estimated with the HRs and 95% CIs derived from a Cox proportional hazards model. The heterogeneity of the randomized treatment effect across apixaban dose categories was tested by adding an interaction to the Cox proportional hazards model. All statistical analyses were performed using SAS System v9.4 (TS1M6) (SAS Institute, Inc).

## Results

Of the 4,614 patients enrolled in AUGUSTUS, 4,549 were included in this analysis and 2,290 received apixaban. A total of 65 patients were excluded from the analysis as they did not start the anticoagulant (apixaban or VKA). Of the 2,290 patients who received apixaban, 229 (10%) received a reduced dose; however, less than one-half met the dose-reduction criteria (Table 1). The number and specific dose-reduction criteria among patients who received a reduced dose of apixaban are shown in Table 2.

**Table 1:** Agreement Between Meeting Dose-Reduction Criteria and Apixaban Dosing^a^

Apixaban Dose Received	Criteria for Dose Reduction Met		Total
	Yes	No	
2.5 mg twice daily	90	139	229
5.0 mg twice daily	8	2,053	2,061
Total	98	2,192	2,290

a Only patients in the apixaban arm receiving at least 1 dose of apixaban are included; 16 patients were randomized to apixaban but never received it (1 met the dose-reduction criteria and 15 did not meet dose-reduction criteria).

**Table 2:** Number and Specific Dose-Reduction Criteria Met

	On Reduced Dose Apixaban	
	Appropriately Reduced	Inappropriately Reduced
Met 0 criteria	0 (0.0%)	99 (71.2%)
Met exactly 1 criterion	0 (0.0%)	40 (28.8%)
Age ≥80 y	0	23
Weight ≤60 kg	0	9
Creatinine ≥1.5 mg/dL	0	8
Met exactly 2 criteria	82 (91.1%)	0 (0.0%)
Age ≥80 y and weight ≤60 kg	45	0
Age ≥80 y and creatinine ≥1.5 mg/dL	33	0
Weight ≤60 kg and creatinine ≥1.5 mg/dL	4	0
Met all 3 criteria	8 (8.9%)	0 (0.0%)

Values are n (%).

### Baseline characteristics

Baseline characteristics by randomized arm and dose-reduction criteria are shown in Table 3. Patients who appropriately received reduced dose apixaban were older, had lower body weight, higher creatinine, and a higher mean CHA_2_SD_2_-VASc score than patients who inappropriately received reduced dose apixaban and those who appropriately received standard dose apixaban.

**Table 3:** Baseline Characteristics by Randomized Arm and Dose-Reduction Criteria

Characteristic	Apixaban Dose			VKA		*P* Value^a^	*P* Value^b^
	2.5 mg Twice Daily		5.0 mg Twice Daily				
	Met DR Criteria (N = 90)	Did Not Meet DR Criteria (N = 139)	Did Not Meet DR Criteria (N = 2053)	Met DR Criteria (N = 100)	Did Not Meet DR Criteria (N = 2,159)		
Age, y	83.4 (81.4, 86.3)	71.1 (64.7, 78.5)	69.9 (63.6, 76.1)	82.4 (81.2, 86.2)	70.4 (63.8, 76.4)	0.31	<0.001
Female	49 (54.4%)	43 (30.9%)	573 (27.9%)	50 (50.0%)	604 (28.0%)	0.54	<0.001
Race						1.00	0.61
White	82 (91.1%)	123 (88.5%)	1,869 (92.1%)	89 (89.9%)	1952 (91.8%)		
Black	0 (0.0%)	3 (2.2%)	26 (1.3%)	0 (0.0%)	29 (1.4%)		
Asian	4 (4.4%)	7 (5.0%)	58 (2.9%)	5 (5.1%)	63 (3.0%)		
Other	4 (4.4%)	6 (4.3%)	76 (3.7%)	5 (5.1%)	83 (3.9%)		
Weight, kg	59.0 (56.0, 73.0)	81.0 (71.0, 90.0)	84.0 (74.3, 95.0)	59.9 (56.4, 76.7)	84.0 (74.9, 95.8)	0.33	<0.001
Serum creatinine, mg/dL	1.5 (0.9, 1.7)	1.0 (0.9, 1.2)	1.0 (0.9, 1.2)	1.5 (1.0, 1.7)	1.0 (0.9, 1.2)	0.81	<0.001
Serum creatinine						0.91	<0.001
<1.5 mg/dL	43 (48.9%)	130 (94.2%)	1,923 (94.4%)	48 (48.0%)	1,990 (92.8%)		
≥1.5 mg/dL	45 (51.1%)	8 (5.8%)	113 (5.6%)	52 (52.0%)	154 (7.2%)		
Creatinine clearance, mL/min	35.5 (29.9, 43.9)	71.5 (55.3, 92.2)	76.8 (58.9, 97.0)	36.8 (31.1, 41.8)	76.9 (59.9, 97.4)	0.88	<0.001
Ever smoked	28 (31.1%)	62 (45.9%)	1,031 (50.5%)	40 (40.8%)	1,077 (50.1%)	0.17	0.03
CHA_2_DS_2_-VASc score	5.3 ± 1.4	3.8 ± 1.4	3.9 ± 1.5	5.4 ± 1.3	3.9 ± 1.5	0.45	<0.001
HAS-BLED score	3.3 ± 0.8	2.9 ± 0.9	2.8 ± 1.0	3.3 ± 0.9	2.8 ± 0.9	0.74	<0.001
Hypertension leading to medication use	83 (92.2%)	115 (82.7%)	1,825 (88.9%)	87 (87.0%)	1,903 (88.1%)	0.24	0.04
Diabetes mellitus	32 (35.6%)	52 (37.4%)	747 (36.4%)	37 (37.0%)	778 (36.0%)	0.84	0.78
Stroke, TIA, or thromboembolism	17 (18.9%)	20 (14.6%)	286 (14.0%)	18 (18.2%)	276 (12.9%)	0.90	0.39
Congestive heart failure, no. (%)	39 (43.3%)	41 (29.5%)	885 (43.1%)	53 (53.0%)	926 (42.9%)	0.18	0.03
Concomitant P2Y12 inhibitor						0.18	0.70
Clopidogrel	86 (95.6%)	129 (92.8%)	1,867 (90.9%)	88 (88.0%)	1,928 (89.3%)		
Ticagrelor	2 (2.2%)	5 (3.6%)	114 (5.6%)	7 (7.0%)	149 (6.9%)		
Prasugrel	0 (0.0%)	2 (1.4%)	25 (1.2%)	0 (0.0%)	24 (1.1%)		
None	2 (2.2%)	3 (2.2%)	47 (2.3%)	5 (5.0%)	58 (2.7%)		
Previous use of oral anticoagulant	43 (47.8%)	67 (48.2%)	1,074 (52.3%)	49 (49.0%)	1,007 (46.6%)	0.87	0.95
On beta-blockers at randomization	63 (70.0%)	92 (66.2%)	1,491 (72.6%)	68 (68.0%)	1,579 (73.1%)	0.77	0.55
On ACE inhibitors/ARBs/ARNI	60 (66.7%)	84 (60.4%)	1,330 (64.8%)	57 (57.0%)	1,405 (65.1%)	0.17	0.34
On diuretics	43 (47.8%)	49 (35.3%)	606 (29.5%)	45 (45.0%)	664 (30.8%)	0.70	0.06
Qualifying index event						0.48	0.01
ACS and PCI	29 (32.2%)	52 (37.4%)	785 (38.4%)	40 (40.0%)	784 (36.5%)		
Medically managed ACS	31 (34.4%)	24 (17.3%)	488 (23.9%)	33 (33.0%)	513 (23.9%)		
Elective PCI	30 (33.3%)	63 (45.3%)	773 (37.8%)	27 (27.0%)	853 (39.7%)		
No. of days from ACS or PCI to randomization	7.7 ± 4.3	6.5 ± 4.4	6.7 ± 4.1	7.6 ± 4.3	6.5 ± 4.2	0.75	0.02
Number of criteria for dose reduction met						0.58	–
0	–	99 (71.2%)	1,631 (79.4%)	–	1,672 (77.4%)		
1	–	40 (28.8%)	422 (20.6%)	–	487 (22.6%)		
2	82 (91.1%)	–	–	94 (94.0%)	–		
3	8 (8.9%)	–	–	6 (6.0%)	–		
Randomized to aspirin	47 (52.2%)	64 (46.0%)	1,030 (50.2%)	47 (47.0%)	1,079 (50.0%)	0.47	0.36

Values are median (25th, 75th), n (%), or mean ± SD.

ACE = angiotensin converting enzyme; ACS = acute coronary syndrome; ARB/ARNI = angiotensin receptor blocker/angiotensin receptor neprilysin inhibitor; DR = dose-reduction; PCI = percutaneous coronary intervention; TIA = transient ischemic attack; VKA = vitamin K antagonist.

a *P* value comparing apixaban met DR criteria vs VKA met DR criteria.

b *P* value comparing apixaban met DR criteria vs apixaban did not meet DR criteria (2.5 mg twice daily dose).

Among patients who met the criteria for reduced dose and received 2.5 mg twice daily (first column in Table 3), 2/90 (2.2%) increased to 5 mg twice daily during follow-up. Among patients who did not meet the criteria for reduced dose and received 2.5 mg twice daily (second column in Table 3), 36/139 (25.9%) increased to 5 mg twice daily during follow-up. Among patients who did not meet the criteria for reduced dose and received 5 mg twice daily (third column in Table 3), 78/2,053 (3.8%) decreased to 2.5 mg twice daily during follow-up.

Overall, the median VKA time in therapeutic range of those patients matched to the apixaban subgroups was 58.6%. This is comparable to the full cohort of the AUGUSTUS trial.^11^ For the standard dose, VKA matched patients the median time in therapeutic range was 58.4%, for the appropriately reduced 61.1%, and for the inappropriately reduced 62.2%.

### Apixaban dose-reduction group and outcomes

**Figure 1:**
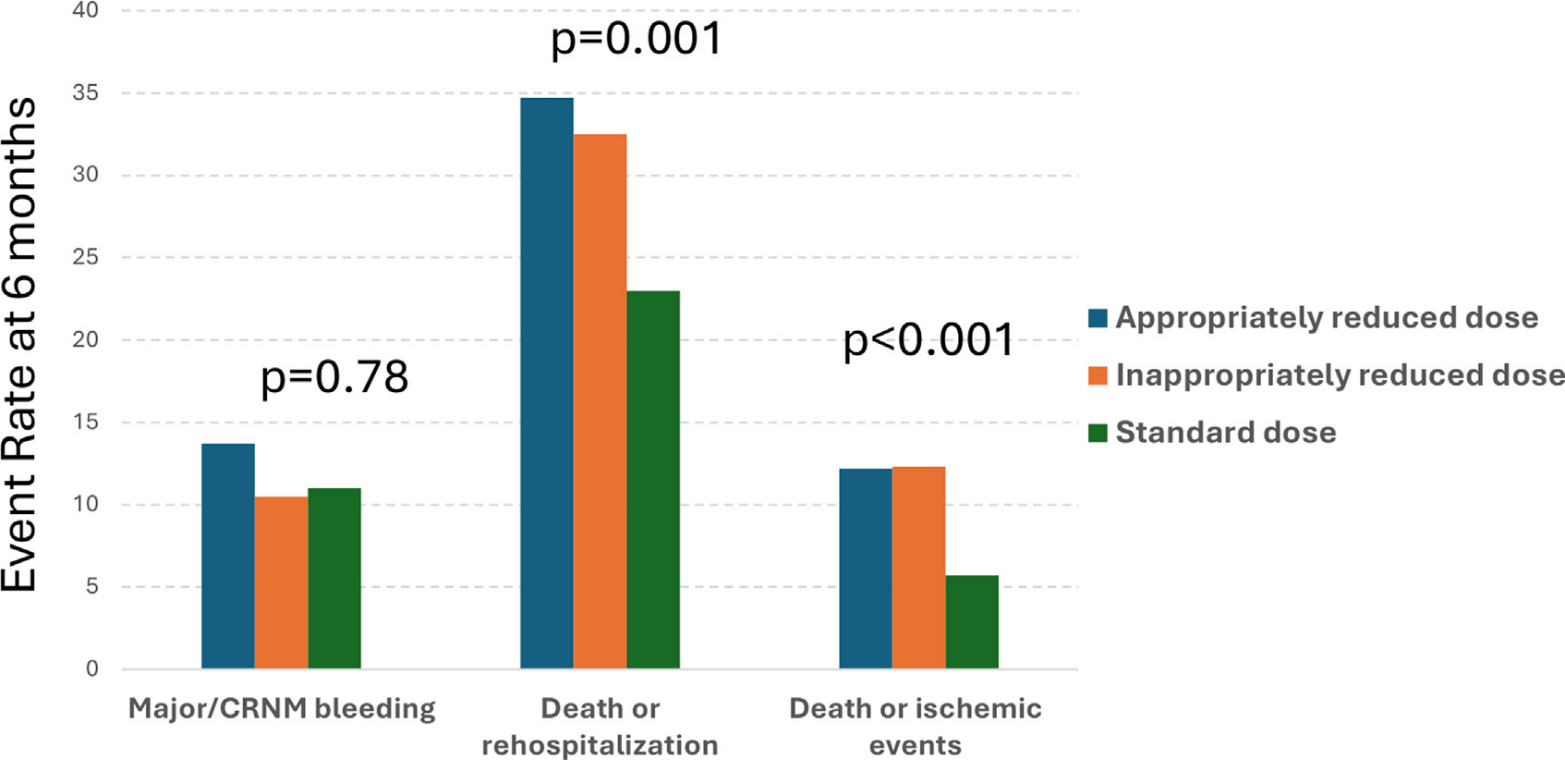
**Apixaban Dose-Reduction Group and Outcomes** Event rates of major/CRNM bleeding, death or rehospitalization, and death or ischemic events. CRNM = clinically relevant nonmajor.

Rates of major/CRNM bleeding, death or rehospitalization, and death or ischemic events were higher in patients appropriately receiving reduced dose apixaban compared with patients inappropriately receiving reduced dose apixaban and patients appropriately receiving standard dose apixaban (Figure 1).

### Comparison of Apixaban vs VKA: effect on outcomes by Apixaban dose-reduction group

**Figure 2:**
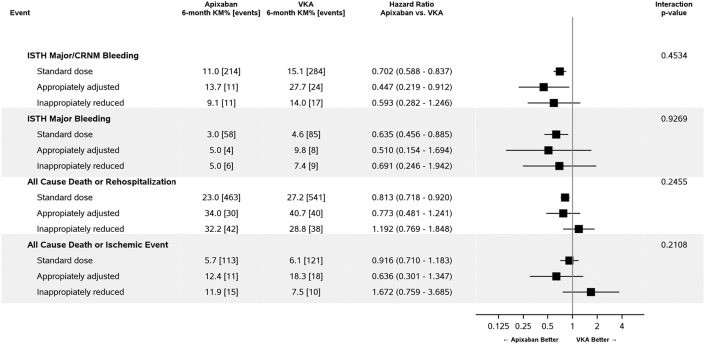
**Randomized Treatment and Clinical Outcomes According to Apixaban Dose** Association between randomized treatment (apixaban vs VKA) and clinical outcomes by standard or reduced dose status (appropriately and inappropriately reduced). ISTH = International Society on Thrombosis and Haemostasis; VKA = vitamin K antagonist; other abbreviation as in Figure 1.

When comparing the risk of all clinical outcomes (major/CRNM bleeding; major bleeding; all-cause death or rehospitalization; all-cause death or ischemic events) among the 3 groups of patients appropriately receiving reduced dose, inappropriately receiving reduced dose, and appropriately receiving standard dose apixaban vs matched patients receiving VKA, we found patients in the apixaban groups to have more favorable outcomes (Figure 2). Patients inappropriately receiving reduced dose apixaban appeared to have less benefit compared with those receiving VKA; however, there was no interaction between any of the groups for any of the tested clinical outcomes (Figure 2).

## Discussion

There are several key findings from this post hoc analysis of the AUGUSTUS trial. First, of the 10% of patients in AUGUSTUS who received a reduced dose of apixaban, less than half met the criteria for dose reduction. Second, patients who received reduced dose apixaban, whether appropriately or inappropriately, were at higher risk of adverse clinical outcomes and bleeding than patients appropriately receiving standard dose apixaban. Third, in each of the 3 apixaban dose groups, the benefits of apixaban compared with VKA were similar, within the limitations of underpowered subgroups analyses. These results suggest that in patients with AF and recent ACS and/or PCI who meet dose-reduction criteria, a reduced dose of apixaban (2.5 mg twice daily) can be safely used.

In the AUGUSTUS trial, patients were assigned to receive apixaban twice daily at a dose of 5.0 mg or 2.5 mg if they met ≥2 or more of the following dose-reduction criteria: age ≥80 years, body weight ≤60 kg, or serum creatinine ≥1.5 mg/dL (133 μmol/L). In our analysis, we observed that of the 10% of patients who received a reduced dose of apixaban, less than half (43%) actually met the criteria for dose reduction; an additional 6.1% of patients who did not meet the criteria for dose reduction also received a reduced dose. The relatively high use of inappropriately reduced dose apixaban is likely explained by concerns about bleeding in patients requiring antiplatelet therapy despite not fulfilling the labeled criteria for dose reduction. Inappropriate reduction could be the result of unmeasured risk factors for bleeding leading investigators to correctly or incorrectly suspect a higher bleeding risk. The observed differences in both bleeding and ischemic outcomes across apixaban dose groups are more likely related to differences in patient characteristics than to the administered apixaban dose. That is why patients appropriately receiving reduced dose apixaban had higher rates of bleeding and death/ischemic events than those receiving standard dose apixaban.

The AUGUSTUS trial showed that, in patients with AF and ACS and/or PCI treated with a P2Y12 inhibitor, apixaban resulted in less bleeding and fewer hospitalizations than VKA and that aspirin resulted in more bleeding than placebo, without any significant difference in ischemic events. The present analysis extends the findings of the AUGUSTUS trial. We observed a significantly lower risk of major/CRNM bleeding and a similar rate of rehospitalization and ischemic events in patients appropriately receiving reduced dose apixaban compared with VKA. Similarly, there was a lower risk of major/CRNM bleeding and rehospitalization and a similar risk of ischemic events in patients appropriately receiving standard dose apixaban compared with VKA. Furthermore, the use of appropriately and inappropriately reduced dose apixaban as well as standard dose apixaban were generally associated with comparable clinical outcomes, with a signal toward less benefit with inappropriately reduced dose apixaban.

The same dose-reduction criteria were used in the ARISTOTLE (Apixaban for Reduction of Stroke and Other Thromboembolic Complications in Atrial Fibrillation) trial,^12^ which demonstrated that apixaban was superior to warfarin for the prevention of stroke or systemic embolism in patients with AF. In ARISTOTLE, a reduced dose of 2.5 mg twice daily (vs standard dose of 5.0 mg twice daily) of apixaban was given to patients who met ≥2 of the dose-reduction criteria in order to minimize the potential for higher drug exposures in a sample that may be at an increased risk of bleeding. Similar effects were observed for stroke or systemic embolism (*P*_interaction_ = 0.22) and major bleeding (*P*_interaction_ = 0.21) when comparing patients who appropriately received reduced dose apixaban with those who appropriately received standard dose apixaban.

A secondary analysis of the ARISTOTLE trial conducted by Alexander et al^13^ sought to determine whether the effects of standard dose apixaban (5 mg twice daily) vs warfarin on stroke/systemic embolism and bleeding varied among patients meeting 1 or none of the dose-reduction criteria. The results demonstrated that patients with isolated older age, low body weight, or renal dysfunction had a higher risk of stroke or systemic embolism and major bleeding and consistent benefits with the standard dose of apixaban vs warfarin when compared with patients without these characteristics. The results of this analysis confirmed the safety and efficacy of standard dose apixaban in patients with AF with 1 dose-reduction criterion, even at the extremes of advanced age, low body weight, and renal dysfunction. Similar dose-reduction strategies were used in the ROCKET AF (Rivaroxaban Once-daily, Oral, Direct Factor Xa Inhibition Compared with Vitamin K Antagonism for Prevention of Stroke and Embolism Trial in Atrial Fibrillation) and ENGAGE AF-TIMI 48 (Effective Anticoagulation with Factor Xa Next Generation in Atrial Fibrillation-Thrombolysis in Myocardial Infarction Study 48) trials.^16^, ^17^, ^18^

In contrast to the dose-reduction strategies used in ARISTOTLE, ROCKET AF, and ENGAGE AF-TIMI 48, the RE-LY (Randomized Evaluation of Long-Term Anticoagulant Therapy)^19^ and ENGAGE AF-TIMI 48 trials^18^ randomized patients to a higher and lower dose of dabigatran and edoxaban, respectively. Both of these trials demonstrated lower rates of bleeding and higher rates of stroke or systemic embolism with the lower dose of oral anticoagulant than the higher dose. To the best of our knowledge, our post hoc analysis of the AUGUSTUS trial is the first to assess the effect of a standard vs reduced dose of apixaban in patients with AF and a recent ACS and/or PCI. Together, the findings of the AUGUSTUS trial and our analysis provide valuable insights that can help clinicians make an informed decision about the optimal dose of antithrombotic therapy in the setting of AF and recent ACS or PCI.

Our findings should be considered in light of a number of limitations. First, this is a post hoc, although hypothesis-driven, nonrandomized secondary analysis of the AUGUSTUS trial which was neither designed nor powered to assess the impact of a reduced (2.5 mg twice daily) vs standard (5.0 mg twice daily) dose of apixaban in patients with AF and a recent ACS and/or PCI. Although the main study was powered for the primary safety outcomes of major or CRNM bleeding, rehospitalizations, mortality, and ischemic events, the results reported for these outcomes in our secondary analysis for the 2 patient subgroups are underpowered and may be insufficient to draw a generalized conclusion. The open-label (yet randomized) treatment with apixaban and VKA may have led to an inadvertent bias. Lastly, the AUGUSTUS trial had specific inclusion and exclusion criteria, limiting the generalizability of these findings to other samples.

## Conclusions

In patients with AF and recent ACS or PCI, appropriate dose reduction of apixaban was associated with a lower risk of bleeding and similar rates of rehospitalization and ischemic outcomes compared with VKA, similar to results found with standard dose apixaban (Central Illustration). Our findings highlight the safety and importance of appropriately applying dose-reduction criteria for apixaban.

**Central Illustration:**
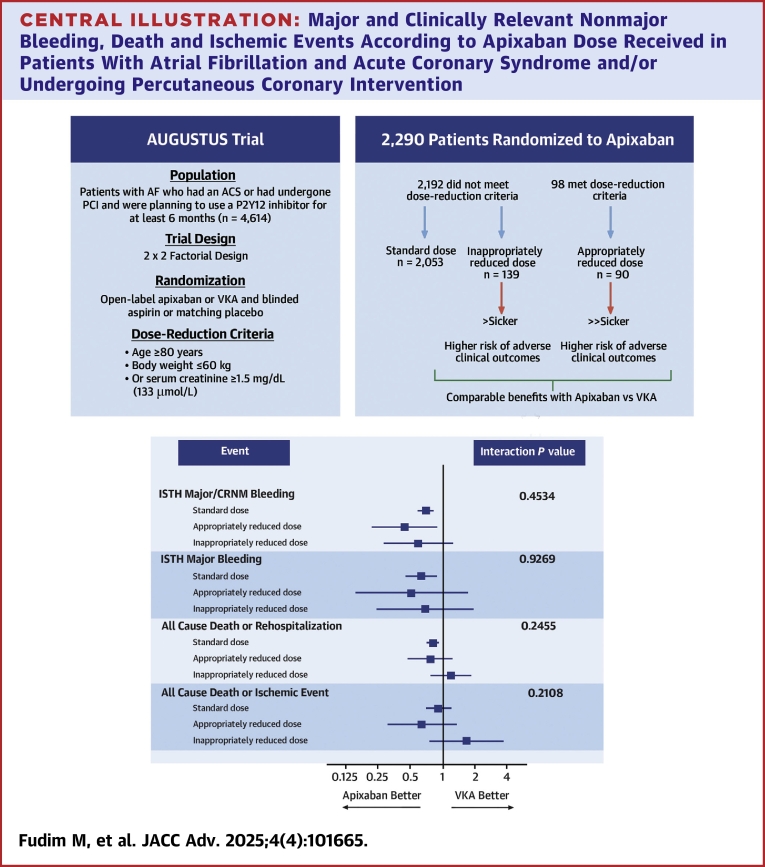
**Major and Clinically Relevant Nonmajor Bleeding, Death and Ischemic Events According to Apixaban Dose Received in Patients With Atrial Fibrillation and Acute Coronary Syndrome and/or Undergoing Percutaneous Coronary Intervention** AUGUSTUS trial sample and association between randomized treatment (apixaban vs VKA) and clinical outcomes by standard or reduced dose status (appropriately and inappropriately reduced). ACS = acute coronary syndrome; AF = atrial fibrillation; PCI = percutaneous coronary intervention; other abbreviations as in Figures 1 and 2.

## Funding support and author disclosures

The AUGUSTUS study and this analysis were funded by Bristol Myers Squibb and Pfizer. Dr Lopes has received research grants or contracts from Amgen, Bristol Myers Squibb, GlaxoSmithKline, Medtronic, Pfizer, and Sanofi-Aventis; has received funding for educational activities or lectures from Pfizer, Daiichi Sankyo, and Novo Nordisk; and has received funding for consulting or other services from Bayer, Boehringer Ingelheim, Bristol Myers Squibb, and Novo Nordisk. Dr Mehran has received institutional research grants from 10.13039/100004325AstraZeneca, 10.13039/100004326Bayer, Beth Israel Deaconess, Bristol Myers Squibb/Sanofi, CSL Behring, Eli Lilly/10.13039/501100022274Daiichi Sankyo, 10.13039/100004374Medtronic, 10.13039/100004336Novartis, and 10.13039/501100018878OrbusNeich; has received consulting fees from Boston Scientific, Abbott Vascular, Medscape, Siemens Medical Solutions, Roivant Sciences Inc, and Sanofi; reports consulting (no fees) for Regeneron Pharmaceuticals Inc; has received institutional consulting fees from Abbott Vascular, Spectranetics/Philips/Volcano Corporation, Bristol Myers Squibb, Novartis, and Watermark Research; is an executive committee member for Janssen Pharmaceuticals and Bristol Myers Squibb; and holds <1% equity in Claret Medical and Elixir Medical. Dr Khan is an advisory board member for Bayer. Dr Granger has received research grants from 10.13039/100004326Bayer, 10.13039/100008349Boehringer Ingelheim, Bristol Myers Squibb, 10.13039/501100022274Daiichi Sankyo, 10.13039/100008897Janssen, 10.13039/100004319Pfizer, Armetheon, 10.13039/100004325AstraZeneca, 10.13039/100000038U.S. Food and Drug Administration, 10.13039/100004330GlaxoSmithKline, 10.13039/100015237The Medicines Company, 10.13039/100016303Medtronic Foundation, 10.13039/100004374Medtronic Inc, 10.13039/100004336Novartis; has received consulting fees from Bayer, Boehringer Ingelheim, Boston Scientific, Bristol Myers Squibb, Daiichi Sankyo, Janssen, Pfizer, Abbvie, Armetheon, AstraZeneca, Eli Lilly, Gilead, GlaxoSmithKline, Hoffmann-La Roche, The Medicines Company, National Institutes of Health, Novartis, Sirtex, Verseon, Apple, Medscape, LLC, Merck, Novo Nordisk, Roche Diagnostics, and Rho Pharmaceuticals. Dr Goodman has received research grant support and/or speaker/consulting honoraria from Amgen, AstraZeneca, Bayer, Boehringer Ingelheim, Bristol Myers Squibb, CSL Behring, Daiichi Sankyo, Eli Lilly, Esperion, Fenix Group International, Ferring Pharmaceuticals, GlaxoSmithKline, HLS Therapeutics, Janssen/Johnson & Johnson, Luitpold Pharmaceuticals, Matrizyme, Merck, Novartis, Novo Nordisk A/C, Pfizer, Regeneron, Sanofi, Servier, Tenax Therapeutics, Heart and Stroke Foundation of Ontario/University of Toronto, Canadian Heart Research Centre and MD Primer, Canadian VIGOUR Centre, Duke Clinical Research Institute, and PERFUSE. Dr Aronson is an employee of Bristol Myers Squibb. Dr Windecker has received institutional research and educational grants from Abbott, Amgen, Bayer, BMS, CSL Behring, Boston Scientific, Biotronik, Edwards Lifesciences, Medtronic, Polares, and Sinomed. Dr Alexander has received research grants from Artivion/Cryolife, Bayer, Bristol Myers Squibb, CSL Behring, Ferring, the U.S. Food and Drug Administration, Humacyte, and the National Institutes of Health; has received consulting fees or honoraria from AbbVie Pharmaceuticals, Artivion/Cryolife, AtriCure, Bayer, Boehringer Ingelheim, Bristol Myers Squibb, Curis, Eli Lilly, Ferring, GlaxoSmithKline, Janssen, Pfizer, Portola Pharmaceuticals, Theravance, and Veralox. All other authors have reported that they have no relationships relevant to the contents of this paper to disclose.
